# ABVD and BEACOPP regimens’ effects on fertility in young males with Hodgkin lymphoma

**DOI:** 10.1007/s12094-020-02483-8

**Published:** 2020-09-17

**Authors:** M. S. A. Amin, O. Brunckhorst, C. Scott, D. Wrench, M. Gleeson, M. Kazmi, K. Ahmed

**Affiliations:** 1 MRC Centre for Transplantation, Guy’s Hospital Campus, King’s College London, King’s Health Partners, London, SE1 9RT UK; 2 Department of Haematology, Guy’s Hospital, London, UK; 3 Department of Urology, King’s College Hospital, London, UK

**Keywords:** ABVD, BEACOPP, Hodgkin lymphoma, Fertility, Young males

## Abstract

**Purpose:**

Considering the increased cancer patient survivorship, the focus is now on addressing the impacts of treatment on quality of life. In young people, altered reproductive function is a major issue and its effects in young males are largely neglected by novel research. To improve clinician awareness, we systematically reviewed side effects of chemotherapy for Hodgkin lymphoma (HL) in young males.

**Methods:**

The review was prospectively registered (PROSPERO N. CRD42019122868). Three databases (Medline via PUBMED, SCOPUS, and Cochrane Library) were searched for studies featuring males aged 13-51-years who underwent chemotherapy for HL using ABVD (Adriamycin® (doxorubicin), bleomycin, vinblastine, and dacarbazine) or BEACOPP (bleomycin, etoposide, doxorubicin, cyclophosphamide, vincristine, procarbazine, and prednisolone) regimens. These chemotherapy regimens were compared against each other using sperm characteristics, FSH, and inhibin B levels to measure fertility levels.

**Results:**

Data were extracted from five studies featuring 1344 patients. 6 months post-ABVD saw marked deterioration in sperm count, further reduced by more cycles (P = 0.05). Patients treated with BEACOPP rather than ABVD were more prone to oligospermia. Receiving fewer cycles of both regimens increased the likelihood of sperm production recovering. Patients treated with 6-8 cycles of BEACOPP did not recover spermiogenesis.

**Conclusions:**

ABVD and BEACOPP regimens significantly reduce fertility function to varying effects depending on treatment duration. ABVD temporarily causes significant reductions in male fertility, whereas BEACOPP’s effects are more permanent. Therefore, clinicians should discuss fertility preservation with male patients receiving infertility-inducing gonadotoxic therapy. Further high-quality studies are required to more adequality describe the risk to fertility by chemotherapy.

**Electronic supplementary material:**

The online version of this article (10.1007/s12094-020-02483-8) contains supplementary material, which is available to authorized users.

## Introduction

Anticancer treatment has become increasingly effective, with strategies possessing high survival rates of around 90% [1, 2]. However, as survivorship improves, the focus has shifted to the impact of treatment on patients’ quality of life with infertility status being one prominent example [1–4]. This is especially relevant in young people where haematological cancers are the most diagnosed malignancies. Reduction in fertility is experienced in 80% of lymphoma patients further emphasized by 20–25% of females encountering difficulties in achieving successful pregnancy [1, 5–7].

These side effects arise largely due to the use of alkylating agent-based chemotherapies, which indiscriminately target the hypothalamic–pituitary–adrenal axis and the gonads. In males, this results in azoospermia, sexual dysfunction, infertility, and associated psychological issues [2, 5, 8–12].

Whilst extensive research has been performed on females in this field, few robust studies exist for males. In response to a recent Cochrane review calling for discussion of treatment-related infertility in males with Hodgkin lymphoma (HL) [13] our study aims to (1) systematically review the literature on effects of anticancer therapy on the fertility of males with HL, (2) identify potential strategies to preserve reproductive function.

## Methods

This systematic review was designed according to the PRISMA Checklist 2009 and prospectively registered via the PROSPERO database, registration number: CRD42019122868 [14].

### Study eligibility criteria

Randomised-control trials, non-randomised studies of interventions, and other observational studies which included males diagnosed with Hodgkin’s lymphoma between the ages 13 and 51 who received anticancer treatment were included in this review. This age range was estimated to be when males are most fertile, between puberty onset and elderly decline in fertility [15–17]. Only studies using ABVD (Adriamycin® (doxorubicin), bleomycin, vinblastine, dacarbazine) or BEACOPP (bleomycin, etoposide, doxorubicin, cyclophosphamide, vincristine, procarbazine, prednisolone) regimens were included as these regimens are the most common and effective treatments for HL [18, 19]. Studies of mixed patients which did not distinguish demographics, interventions, or without pre-treatment and post-treatment data were excluded. Additional exclusion criteria were studies published before January 2000, not written in English, conference abstracts, reviews, editorials, case reports or series.

### Information sources and search

Three databases were searched; MEDLINE via PubMed, Scopus and Cochrane Library up to 27 May 2020, filtering for human studies, written in English and published after 01/01/2000. A combination of search terms and keywords was utilised with variations in spelling also considered. Key words used were “MALE”, “HODGKIN LYMPHOMA” “LYMPHOMA”, “FERTILITY”, “INFERTILITY”, “TREATMENT”, in combinations using Boolean operators (Appendix A). ClinicalTrials.gov and the ISRCTN registry were searched for ongoing clinical trials with authors contacted for unpublished results.

### Study selection

Selection of studies was carried out independently by two reviewers (MSAA and CS). Following deduplication, titles and abstracts were screened and potentially relevant articles selected for full-text retrieval. Reasons were stated for excluding articles after full-text review. Discrepancies between authors were reviewed by a third author (OB) until 100% agreement was achieved.

### Data collection and data items

Data extraction was performed independently by MSAA and CS, again with OB reviewing any inconsistencies. Study characteristics extracted included, author, year, population description, number of participants, study aim, design, treatments received, and author conclusions.

Primary outcomes extracted were measures assessing the chemotherapy-induced changes to fertility. This was measured using sperm count, spermatogenesis, sperm motility and changes to gonadal function reflected by concentrations of sex hormones (follicle stimulating hormone (FSH), luteinizing hormone (LH), Inhibin B, and testosterone). Secondary outcomes for this review were methods for fertility preservation using identical measures to our primary outcome to assess their effectiveness.

### Risk of bias assessment

All selected studies were critically appraised for risk of bias by two authors (MSAA and CS). As no RCTs were identified the ‘Risk Of Bias In Non-randomized Studies of Interventions’ (ROBINS-I) tool was utilised [20]. Studies which scored an overall rating of “CRITICAL” were not taken forward for data extraction or discussion.

## Results

### Study selection and risk of bias

**Fig. 1:**
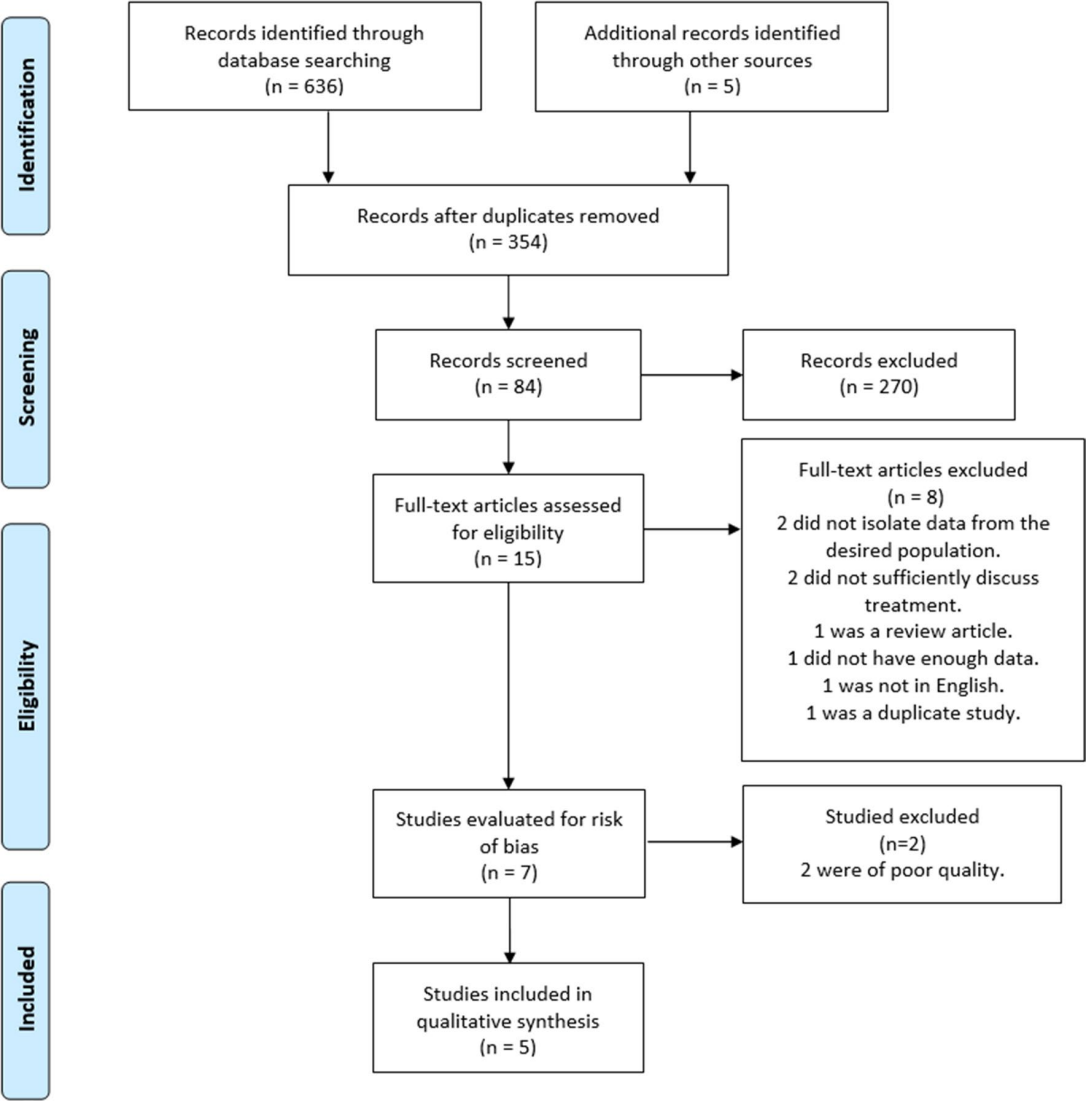
PRISMA flow diagram of studies screened, excluded, and included

Initial searches yielded 636 articles, with 354 remaining post deduplication (Fig. 1). After title and abstract screening 15 articles were reviewed in full of which 7 were selected for critical appraisal using the ROBINs-I test (Appendix B). All studies scored ‘moderate’ for risk of bias in measurement outcomes due to a lack of blinding between patient groups and assessors. Two studies scored ‘critical’ for overall risk study bias and were excluded [21, 22] leaving 5 with a ‘low’ score and therefore suitable for final inclusion and data analysis (Table 1) [4, 23–26].

**Table 1:** Study characteristics

First author, year	Population description	No. of relevant participants	Study aim	Study design
Tal, 2000 [23]	Males with HL 16–42 years old	25	Evaluate changes to semen characteristics due to: chemotherapy, disease characteristics	Cohort
Sieniawski, 2008 [24]	Males with HL 16–41 years old	38	Evaluate fertility changes due to: chemotherapy	Cohort
O’Flaherty, 2010 [25]	Males with HL 21–48 years old	16 cancer patients 11 health controls	Evaluate changes to semen characteristics due to: chemotherapy	Cohort
Behringer, 2013 [4]	Males with HL 18–49 years old	761	Evaluate gonadal changes due to: chemotherapy	Cohort
Paoli, 2016 [26]	Males with HL 13–51 years old	504	Evaluate changes to semen characteristics due to: chemotherapy, age, disease characteristics	Retrospective cohort

*HL* Hodgkin lymphoma

### Study characteristics

Together, the 5 studies discussed 1344 males with HL and comprised of 1 retrospective cohort [26] and four prospective cohort studies [4, 23–25] (Table 2). All studies discussed chemotherapy for HL with four featuring ABVD [4, 23, 25, 26], two featuring BEACOPP [4, 24], and three featuring escBEACOPP [4, 24, 26]. Three studies measured semen characteristics and FSH levels [23–25], two studies measured only semen characteristics [25, 26] and one study used Inhibin B/FSH ratios [4].

**Table 2:** Study outcomes

First Author, Year	Treatment received	Outcome measures	Results	Conclusion
Tal, 2000 [23]	ABVD	Sperm characteristics: count Sex hormone levels: FSH, testosterone	33% of patients became azoospermic post-treatment. Pre-treatment testosterone and FSH concentrations gave no prediction of this change (*P* > 0.05) Sperm concentration reductions were more pronounced with advanced disease stage due to enhanced treatment, rather than the disease itself (*P* = 0.016)	ABVD is sufficiently gonadotoxic
Sieniawski, 2008 [24]	BEACOPP (baseline), Escalated BEACOPP	Semen volume Sperm characteristics: count, concentration, motility, morphology Sex hormone levels: LH, FSH, testosterone	89% of post-treatment patients had azoospermia and 11% had dyspermia 11% of patients recovered spermatogenesis between 1.5–6.7 years post-therapy There was no statistical significance in infertility rates between baseline and escalated BEACOPP post treatment (*P* > 0.999) There were significant differences between pre and post treatment FSH (*P* = 0.008) but not for LH (*P* = 0.203) or testosterone (*P* = 0.844)	Risk to fertility in HL patients is regime dependent FSH is useful in measuring male fertility
O’Flaherty, 2010 [25]	ABVD	Sperm characteristics: concentration, motility, morphology Sex hormone levels: FSH	Pre-treatment sperm quality was similar in HL and control groups At 6 months post-treatment, 40% were azoospermic 38% oligozoospermic with significant decrease in normal sperm forms At 12 months, normal sperm form recovered At 12–18 months, 50% of patients had recovered At 18 months, motility returned to normal At 24 months, 6% were oligozoospermic At 24 months, 57% were normospermic FSH values were only low at 6 and 12 months post-therapy when compared to healthy controls	Survivors of post-chemotherapy
Behringer, 2013 [4]	ABVD, BEACOPP (baseline), Escalated BEACOPP	Sex hormones: Inhibin B/FSH ration	Post-treatment Inhibin B/FSH ratios were closer to fertile levels in early stage patients when compared to the advanced stage (*P* < 0.001) Spermatogenesis did not recover in patients treated for advanced stage HL Patients treated with BEACOPP were more likely to have oligospermia Patients treated for early stage disease significantly more likely to give birth to children via natural fertilization than late stage patients (*P* = 0.04)	Chemotherapy is gonadotoxic Fertility preservation is required
Paoli, 2016 [26]	ABVD + radiotherapy, Escalated BEACOPP	Sperm characteristics: count, concentration, motility, morphology	Post-treatment semen count remained in normal ranges. However, early stage disease had a higher count compared to late stage ABVD (2–8 cycles) with inguinal sparing IF RT significantly decreases total sperm number at 6 months (*P* = 0.001) and 12-months (*P* = 0.01) post-treatment At 6-months sperm motility was reduced (*P* = 0.001) and abnormal forms increased (*P* = 0.01). No significant differences found between 0 and 24-months post-treatment Patients treated with lower number of treatment cycles had higher sperm at 6 months (*P* = 0.05). Spermatogenesis was only impacted by number of treatment cycles, not age or disease stage (*P* = 0.05) All patients who received ABVD (2–6 cycles) + inguinal RT were azoospermic at 6 months. Recovery of spermiogenesis took up to 5 years with sperm quality being severely impaired Patients who received BEACOPP (2–4 cycles) recovered spermatogenesis post 3–4 years post-treatment. Spermatogenesis did not recover when given BEACOPP (6–8 cycles)	Pre-treatment normozoospermia found in 75% of patients Recovery of spermatogenesis is regime dependant

ABVD: doxorubicin, vinblastine, dacarbazine, bleomycin

BEACOPP: bleomycin, etoposide, doxorubicin, cyclophosphamide, vincristine, procarbazine, prednisone

IFRT: involved field radiotherapy

FSH: follicle stimulating hormone

LH: luteinising hormone

### Changes to sperm count

Pre-treatment sperm count was higher in early stage disease when compared to late stage but was still in normal ranges [23, 26]. Subsequent changes to these pre-treatment values were verified by interval or regression analysis to be strictly due to treatment used and not age or disease stage (*P* = 0·05) [4, 23, 26]. However, one study observed no difference in pre-treatment sperm quality between HL or control group patients [25].

At 6 months post-ABVD, 38% of patients had oligospermia, with a further 40% having azoospermia [25]. 6–8 cycles of ABVD lowered sperm count more than 2–4 cycles (*P* = 0·05) [26]. ABVD combined with radiotherapy, either inguinal sparing or inguinal involved field caused patients to become oligospermic or azoospermic, respectively at 6 months [26]. A minority of patients who had received inguinal radiotherapy recovered spermiogenesis but sperm quality was severely impaired [26]. Inguinal-sparing radiotherapy maintained oligospermia in patients at 12 months post-treatment (*P* = 0·01) [26]. By 12–18 months, 50% of patients treated with ABVD had recovered normal sperm characteristics [4] which rose to 57% at 24 months with Paoli et al. observing normospermia by 24 months [25, 26].

Paoli et al. [26] reported three patients who underwent 2–4 cycles of BEACOPP recovered sperm function by 4 years post-treatment, whereas 13 patients who received 6–8 cycles BEACOPP did not. Behringer et al. [4] also observed that patients who had 6–8 cycles of BEACOPP did not recover spermiogenesis.

There were no statistical differences in fertility rates between males treated with BEACOPP or escBEACOPP regimen (*P* > 0·999) with 89% becoming azoospermic and 11% dyspermic [24]. Only 4% of dyspermic patients recovered spermatogenesis with timing varying from 1.5–6.7 years post-therapy [24].

Patients treated with BEACOPP were more likely to have oligospermia than those treated with ABVD [4]. As such, patients with early stage HL were significantly more likely to have children born via natural methods due to the more gonadotoxic treatment used for advanced stage disease [4].

### Changes to sperm morphology

At 6 months, patients treated with both ABVD and inguinal-sparing radiotherapy had significantly decreased sperm motility (*P* = 0.001) and significant changes to sperm morphology (*P* = 0.01) when compared to pre-treatment values [26]. O’Flaherty et al. [25] also recorded a decrease in the proportion of sperm demonstrating normal physiology at 6 months in patients treated solely with ABVD.

### Changes to sex hormones

Pre-treatment values of serum testosterone and FSH gave no indication of post-therapy azoospermia (*P* > 0.05) [23]. Pre-treatment Inhibin B/FSH ratios were closer to fertile levels in early stage rather than late stage disease [4]. FSH was raised at 6 and 12 months post-ABVD treatment compared to healthy controls (*P* = 0.008) before returning to normal [4, 23, 25]. Treatment using BEACOPP (6–8 cycles) resulted in lower Inhibin B/FSH ratios corresponding to levels of impaired fertility (*P* < 0.001) [4]. LH and testosterone did not significantly change before and after treatment with ABVD (*P* = 0.203, *P* = 0.844, respectively) [23].

### Fertility preservation

None of the studies addressed fertility preservation methods for patients with HL so data on successful pregnancies was not extracted. However, it was reported that natural conception of offspring post-treatment was more likely in patients with early stage disease than advanced (*P* = 0.04) [4].

## Discussion

Fertility risk is important to young adult male patients, yet clinicians are uncomfortable with discussing this issue as they are ill-informed to side effects of anticancer treatment [2, 8, 27, 28]. Consequently, patients are unable to make informed treatment decisions surrounding their fertility and potential reproductive function post-treatment. The ensuing inability to conceive can result in anxiety and depression for patients [28]. Experts recommend that patients wishing to preserve fertility be referred to specialists [11] or be informed of preservation methods before commencing treatment [4]. However, it has been reported that more than 50% of oncologists do not refer their young adult patients for fertility preservation, going against the American Society of Clinical Oncology (ASCO) guidelines [27]. Raising awareness of side effects of anticancer treatment to male fertility may promote referral to underutilised fertility preservation services [8, 11]. This review highlights the high impact of ABVD and BEACOPP chemotherapy on fertility in males with Hodgkin lymphoma, and thereby the need for utilisation of these services.

ABVD is used in patients with early and advanced stage HL [18, 29]. In patients who took ABVD, normal reproductive function returned between 12 months and 2 years post-treatment as reflected by rises in sperm count and serum FSH levels. Furthermore, increased number of ABVD cycles from 2–4 to 6–8 is associated with marked reductions to sperm count, delaying and reducing recovery of reproductive function and more unlikely to regain normospermia. This demonstrates that longer exposure to gonadotoxic material has an incremental impact on fertility. Patients who received ABVD alongside inguinal sparing radiotherapy recovered normospermia by 2 years post-remission. On the other hand, patients who received ABVD but with inguinal radiotherapy took up 5 years to recover spermiogenesis while sperm motility and morphology remained impaired. As lymphoma management may use variations of adjuvant radiotherapy e.g. inguinal or inguinal sparring [5], the associated risks to quality of life should be discussed with the patient.

The BEACOPP regimen is used for the treatment of advanced stage HL [18]. However, despite having a better progression-free survival rate than ABVD, the presence of enhanced alkylating agents makes it more gonadotoxic than ABVD increasing the risk of oligospermia and resultant lower quality of life [2, 4, 5, 10, 30]. Similarly, to the increased cycles of ABVD, 6–8 cycles of BEACOPP resulted in no recovery of reproductive function, whereas it did return 3–4 years post-treatment when 2–4 cycles were used. Ultimately, BEACOPP results in great damage to spermiogenesis, with most patients never recovering function.

As serum FSH levels mirrored changes to sperm count, serum FSH may have potential as a convenient early marker of post-treatment fertility. Changes to other sex hormones were not reflective of altered gonadal function.

The secondary outcome was to evaluate effective methods of fertility preservation. No methods were identified yet it remains an important factor for discussion. Recommendations for fertility preservation by the American Society of Clinical Oncology (ASCO) and the European Society of Medical Oncology (ESMO) include sperm cryopreservation which can be used for intrauterine insemination [11, 28]. Other artificial reproduction technologies include gonadal stem cell transplants and testicular tissue preservation, but as they are untested, sperm cryopreservation remains the best option for sperm-producing males [3, 31–33]. However, only a few studies identify the effectiveness of this method in producing children, but this may be due to patients being of a young age, having no plans for offspring, or lost to follow-up [3].

**Fig. 2:**
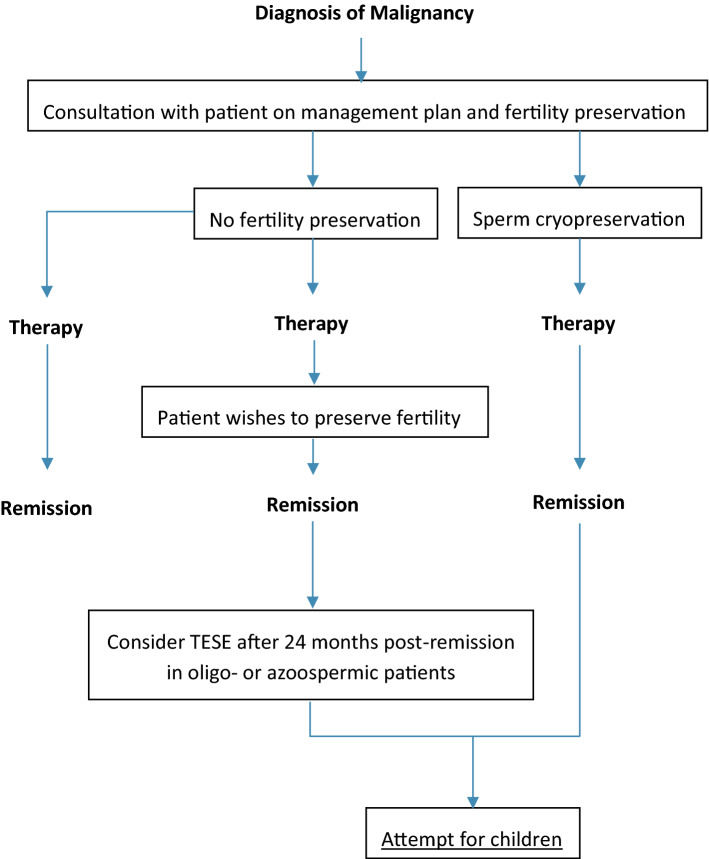
Flow diagram of suggested fertility preservation management. TESE: testicular sperm extraction

One study not included in the review found microsurgical testicular sperm extraction (TESE) beneficial in retrieving sperm from young males with leukaemia and Hodgkin lymphoma who did not cryopreserve their sperm prior to treatment identifying its clinical potential [34]. Guidance from the American Society for Reproductive Medicine (ASRM) advocates TESE usage in oligo- or azoospermic males [3, 33, 35]. However, evidence not included in the guidance suggests waiting 18–24 months post-therapy to allow for the testes to be cleared of damaged germ cells before seeking artificial reproductive methods [31, 36]. Despite lacking a high-quality evidence for TESE in post-treatment fertility preservation, we have used the guidance from these leading clinical societies and the methods used for other cancers, to suggest fertility management strategies for Hodgkin lymphoma patients if sperm cryopreservation fails (Fig. 2).

This review has served to identify the impacts of treatments of common haematological malignancy treatment for young males where gaps in the literature exist. The review’s primary strength was the robust methodology including only relevant content, with a low risk of bias as identified by the ROBINS-I score. The large populations and homogenous results allowed for study comparison and development of common conclusion. However, as with any review, there are limitations. First, the review was limited by the relatively small number of articles included. Moreover, whilst articles included had similar aims in observing sperm characteristics, there was heterogeneity in exact measures recorded. Studies which measured the same outcomes observed different exact treatments or released raw data from incomparable categories, thereby preventing any meaningful meta-analysis from being conducted. Furthermore, due to human error there is always the risk of relevant studies being missed during the literature search; however, we aimed to minimise this by our comprehensive methodology using independent reviewers. Finally, there is a limitation within the overall level of evidence included as no randomised studies were suitable for inclusion.

To address the limitations of the review and of the literature, high-quality randomised controlled trials are certainly required to further define the fertility implications of cancer treatment in Hodgkin lymphoma. Treatment regimens must be precisely documented with outcomes measuring pre-treatment fertility markers such as sperm count, sperm motility, and FSH levels. Subsequent samples should be taken at 0, 6, 12, 18, and 24 months post-treatment for comparison with significant endpoints such as pregnancy rates also accommodated for.

## Conclusion

**Fig. 3:**
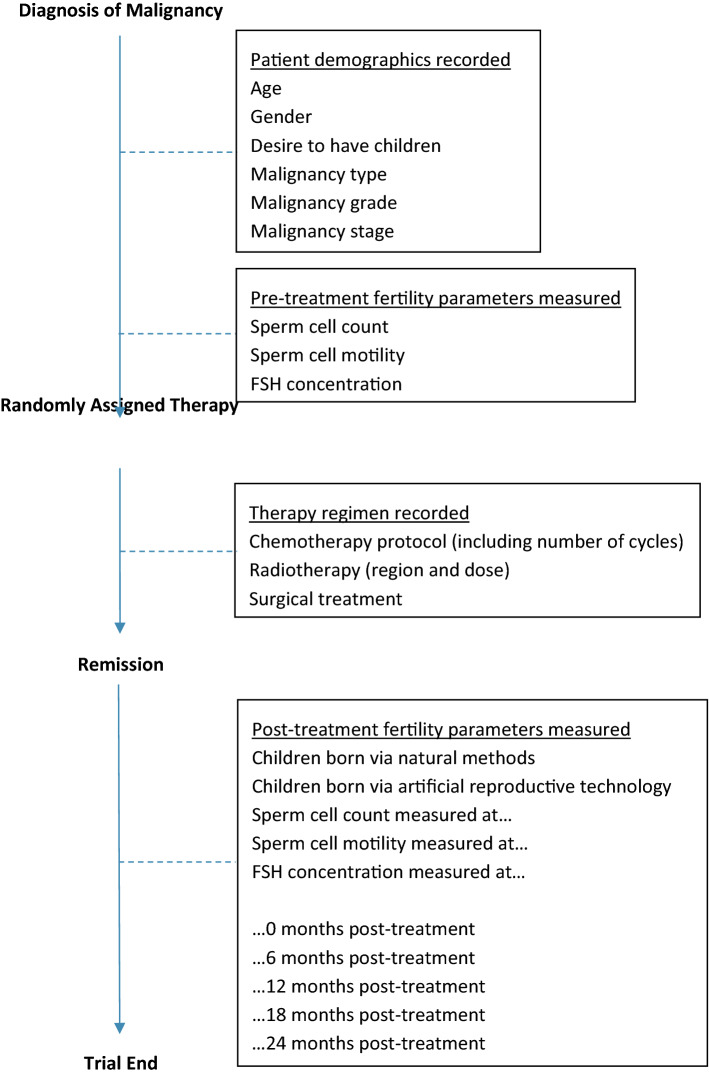
Flow diagram of recommendations for future fertility studies

The literature review demonstrates that ABVD and BEACOPP treatments are gonadotoxic in males with Hodgkin lymphoma. Strong evidence suggests that ABVD is the less gonadotoxic option of the two regimens and prolonged treatment cycles lead to greater gonadal insult culminating in permanent infertility. Therefore, clinicians ought to adequately discuss fertility risk and management options with patients prior to commencing these treatments and potentially other alkylating agent-based regimens. Future clinical practice may involve modifications to established regimens as it has been suggested that this may reduce therapy side effects [37]. One example of a modification is BEACOPP being changed to BEACOP-Dac where dacarbazine replaces procarbazine. This novel de-escalation therapy aims to be less sterilising by giving fewer courses of chemotherapy to patients who achieve complete response to the original regimen, thereby reducing the side effects to fertility. However, this is yet to be evaluated. At present, only limited evidence exists for fertility preservation options pre- and post- treatment. However, we have extrapolated methods from study designs to recommend a research pathway for future studies to effectively focus on clinically important outcomes including side-effects of treatments and effective methods for fertility preservation (Fig. 3). In the absence of these studies, the conclusions of this review regarding HL treatment may be used as a basis for future fertility preservation in young males treated for other forms of cancer.

## Electronic supplementary material

Below is the link to the electronic supplementary material.

Supplementary file1 (DOCX 557 kb)
