# Supplementary material for: ABVD and BEACOPP regimens’ effects on fertility in young males with Hodgkin lymphoma
Source: Clin Transl Oncol. 2020 Sep 17;23(6):1067–77. doi: 10.1007/s12094-020-02483-8 (PMC8084804; doi:10.1007/s12094-020-02483-8)
Supplement: Supplementary file 1 — Supplementary file1 (DOCX 557 kb) [file 12094_2020_2483_MOESM1_ESM.docx]

**APPENDIX A: SEARCH STRATEGY AND FIGURES**

Male AND lymphoma AND fertility AND treatment

PubMed – 162 (searches from 01/01/2000 – 27/05/2020)
Scopus – 168 (Limit articles to English, article, searches from 01/01/2000 – 27/05/2020)
Cochrane Library – 1
Total - 331

Male AND lymphoma AND infertility AND treatment

PubMed – 155 (searches from 01/01/2000 – 27/05/2020)
Scopus – 170 (Limit articles to English, article, searches from 01/01/2000 – 27/05/2020)
Cochrane Library – 1
Total - 326

Male AND Hodgkin lymphoma AND fertility AND treatment

PubMed – 103 (searches from 01/01/2000 – 27/05/2020)
Scopus – 92(Limit articles to English, article, searches from 01/01/2000 – 27/05/2020)
Cochrane Library – 1
Total - 196

Male AND Hodgkin lymphoma AND infertility AND treatment

PubMed – 106 (searches from 01/01/2000 – 27/05/2020)
Scopus – 94 (Limit articles to English, article, searches from 01/01/2000 – 27/05/2020)
Cochrane Library – 1
Total - 201

**APPENDIX B: RISK OF BIAS**

| **First Author, Year** | **Bias due to  confounding** | **Bias in selection of participants into the study** | **Bias in classification  of interventions** | **Bias due to deviations from intended interventions** | **Bias due to missing data** | **Bias in measurement  of outcomes** | **Bias in selection of  the reported result** | **Overall risk of bias** |
| --- | --- | --- | --- | --- | --- | --- | --- | --- |
| Paoli et al, 2016 [26] | Low | Low | Low | Low | Low | Moderate | Low | Low |
| Boltezar et al, 2016 [21] | Critical | Moderate | Serious | Critical | Low | Moderate | Low | Critical |
| Behringer et al, 2013 [4] | Low | Low | Low | Low | Low | Moderate | Low | Low |
| O'Flaherty et al, 2010 [25] | Low | Low | Low | Low | Low | Moderate | Low | Low |
| Sieniawski et al, 2008 [24] | Low | Low | Low | Low | Low | Moderate | Low | Low |
| Van der Kaaij et al, 2007 [22] | Critical | Moderate | Critical | Critical | Moderate | Moderate | Low | Critical |
| Tal et al, 2000 [23] | Low | Low | Low | Low | Low | Moderate | Low | Low |
